# Scattering of Attosecond Laser Pulses on a DNA Molecule during Its Nicking and Bending

**DOI:** 10.3390/ijms242115574

**Published:** 2023-10-25

**Authors:** Dmitry Makarov, Anastasia Kharlamova

**Affiliations:** Department of Fundamental and Applied Physics, Northern (Arctic) Federal University, Nab. Severnoi Dviny 17, 163002 Arkhangelsk, Russia; a.a.harlamova@narfu.ru

**Keywords:** X-ray, DNA, X-ray diffraction, attosecond pulse, USP, nick (DNA), bending DNA

## Abstract

It is well known that X-ray crystallography is based on X-ray diffraction (XRD) for atoms and molecules. The diffraction pattern arises as a result of scattering of incident radiation, which makes it possible to determine the structure of the scattering substance. With the advent of ultrashort radiation sources, the theory and interpretation of X-ray diffraction analysis have remained the same. This work shows that when an attosecond laser pulse is scattered on a DNA molecule, including during its nicking and bending, the pulse duration is an important characteristic of the scattering. In this case, the diffraction pattern changes significantly compared to the previously known scattering theory. The results obtained must be used in XRD theory to study DNA structures, their mutations and damage, since the previously known theory can produce large errors and, therefore, the DNA structure can be “decoding” incorrectly.

## 1. Introduction

It is generally believed that when scattering ultrashort laser pulses (USP) in the X-ray frequency range, the results do not depend on the duration of such a pulse τ. As a result, the theory of diffraction analysis of matter (XRD) usually uses a previously known theory based on an infinitely long pulse duration [1,2,3,4]. In this theory, the key quantity in X-ray scattering is the scattering spectrum (scattered energy of incident radiation into a solid angle), related to the Fourier transform of the electron density distribution ρ(r) in a substance as follows: [5,6]

(1) $$\begin{array}{c} \frac{d\varepsilon }{d\Omega_{k}}=\frac{d\varepsilon_{e}}{d\Omega_{k}}{\left|\rho \left(r\right)e^{ipr}d^{3}r\right|}^{2}, \end{array}$$

where $\frac{d\varepsilon_{e}}{d\Omega_{k}}$ is the scattering spectrum (scattered energy into a solid angle by one electron) of a free electron (Thomson scattering), p is the momentum transferred to the electron during scattering (otherwise $p=\frac{2\pi }{\lambda }\left(n-n_{0}\right)$ is the scattering vector, where n is the direction of scattered radiation, $n_{0}$ is direction of incident radiation) and Ω is the solid angle at which scattering occurs. Using well-known methods [7] based on the inverse Fourier transform, one can determine ρ(r) from the X-ray scattering pattern. It is also believed that ultrashort sources of laser pulses are important, primarily for studying the dynamics of processes occurring in various structures [8,9,10,11]. In this case, the interaction time of such pulses τ with the structure under study should be many times lower than its characteristic time $\tau_{a}$, i.e., $\tau \ll \tau_{a}$. In other words, to find the electron density varying with time, you need to find ρ(r,t), i.e., in Equation (1), you need ρ(r)→ρ(r,t) [6]. This method is well known as time-resolved X-ray diffraction (TR-XRD). This method is, in fact, a generalization for many of today’s approaches, where one can observe visualization in four dimensions x,y,z,t. In other words the TR-XRD technique uses the same Equation (1), but the diffraction pattern is “read off” the system being studied over time *t* and conclusions are drawn about the dynamics of the system from a large set of such patterns. Measuring the time dynamics of such processes includes two stages. The first is the launch of the dynamic process under study (“pumping” the sample), and the second is the collection of diffraction patterns with different time delays through ultrasonic irradiation of the system under study (sample probing, i.e., “probe”). This method is well known as “pump-probe”. To study such processes, high-power USPs are used, since to study dynamic processes with characteristic times $\tau_{a}$, USPs of even shorter duration τ are required, i.e., the condition $\tau \ll \tau_{a}$ must be satisfied. A very powerful USP source is needed because in the short time τ of interaction between the USP and the system under study, enough radiation has been scattered so that it can be detected. To implement such a concept, difficulties arise due to the destruction of the test sample due to the high power of the USP. This problem can already be solved for femtosecond pulses, since the structure is destroyed over a much longer time, i.e., The USP “reads” the information when the USP is scattered, and only then the structure is destroyed [12,13]. Of course, it is possible to study the structure without resorting to ultrashort pulses, but only in the case of stationary objects. If dynamic structures are to be studied, ultrashort pulses must be used, and the theory of scattering of such pulses for the study of dynamic structures must contain the pulse duration parameter, and it is incorrect to use Equation (1) directly. One way or another, the problem of interaction of ultrashort pulses with atoms and molecules is an important part of modern physics [6,9,14,15,16,17].

Recently, in the works in [18,19], it was shown that Equation (1) is not correct to use in the case of scattering of attosecond laser pulses on nucleotides and trinucleotides of DNA and RNA. It was shown that the previously known XRD theory can differ greatly from the case of the theory developed in these works, which takes into account the USP duration parameter. Extending similar conclusions to more complex DNA structures, for example, their mutations, breaks, folding, etc., is not possible directly. This is due to the fact that only direct calculation and analysis of a specific structure using the theory in [18] can provide information about the effect of pulse duration on scattering spectra. It can only be argued that such pulses should be attosecond and shorter when scattered on biomolecules [18,19].

**Figure 1:**
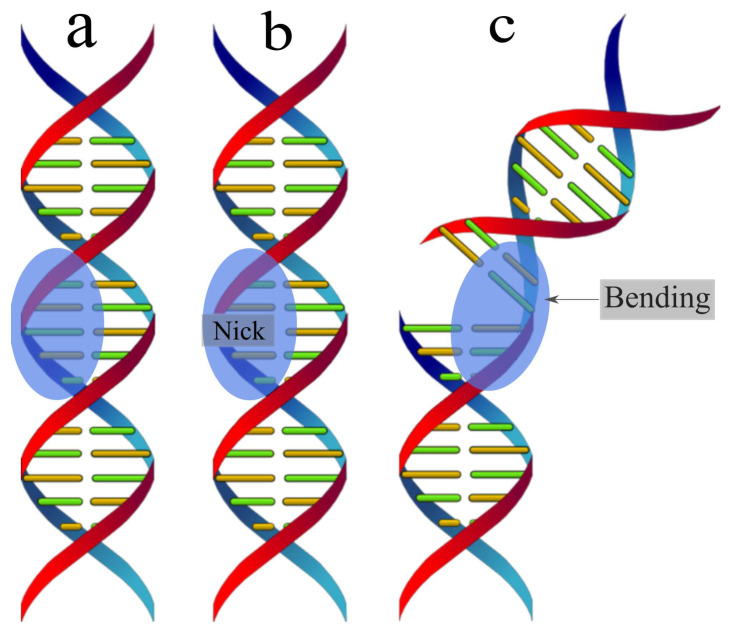
(**a**) a DNA molecule is shown; (**b**) a DNA molecule is shown with a break in which there is no phosphodiester bond between adjacent nucleotides, i.e., Nick; (**c**) bending DNA in the Nick molecule. The highlighted areas in the figures are the non-periodic part of the DNA molecule and are of interest for calculating scattering spectra.

A single-stranded DNA macromolecule with five nitrogenous bases was chosen as the object of study: cytosine–cytosine–guanine–cytosine–cytosine. It is interesting to consider a few states of the molecule that are important in biology, namely the stacking interactions of DNA with Nick—the absence of a single phosphodiester bond—bending DNA, see Figure 1. Nick can be either mutational damage or the result of a directed biochemical reaction [20]. Nick allows DNA to unwind during replication, is a marker for ligase recognition and was detected via XRD, at 2A resolution [21] using Equation (1). To achieve higher resolution, the results obtained were refined using iterative single isomorphic substitution methods to obtain electron density maps, also via heavy atom methods [22], and indirectly by detecting the embedded ligase in place of Nick [23]. The obtained data do not give a clear understanding of the structure of the studied sample, which is reflected in the search for new ways to find Nick in the DNA structure [24].

This work shows that the previous XRD method gives a large error in the USP scattering spectra in the case of using attosecond pulses on the objects under study, which can lead to incorrect interpretation and “decoding” of these structures.

Next, the atomic system of units is used, *ℏ* = 1; |e| = 1; $m_{e}$ = 1, where *ℏ* is the Dirac constant, *e* is the electron charge and $m_{e}$ is the electron mass.

## 2. Results

**Figure 2:**
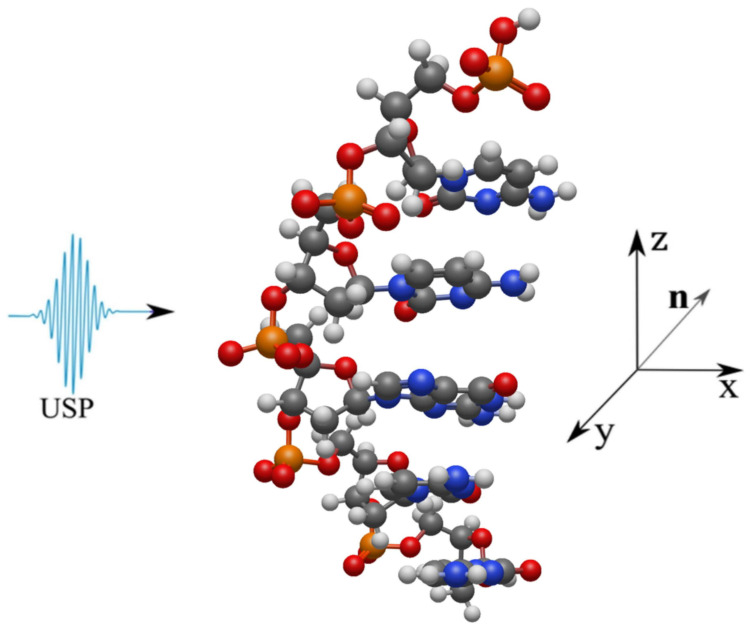
The USP is shown falling on a section of a single-stranded DNA molecule. The red balls are oxygen atoms (*O*), the yellow ones are phosphorus (*P*), the blue ones are nitrogen (*N*), the light grey ones are hydrogen (*H*) and the dark grey ones are carbon (*C*).

**Figure 3:**
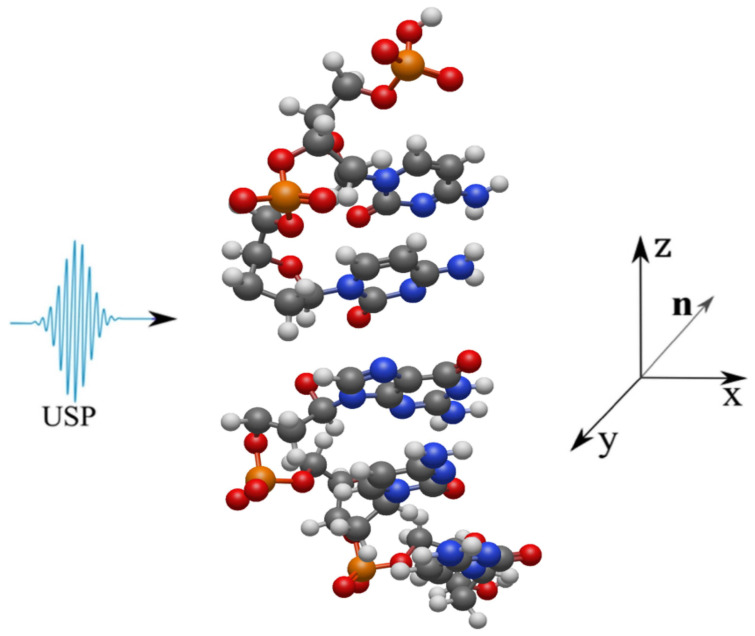
The USP is shown falling on a section of a single-stranded Nick molecule. The colors of the atoms are the same as in Figure 2.

**Figure 4:**
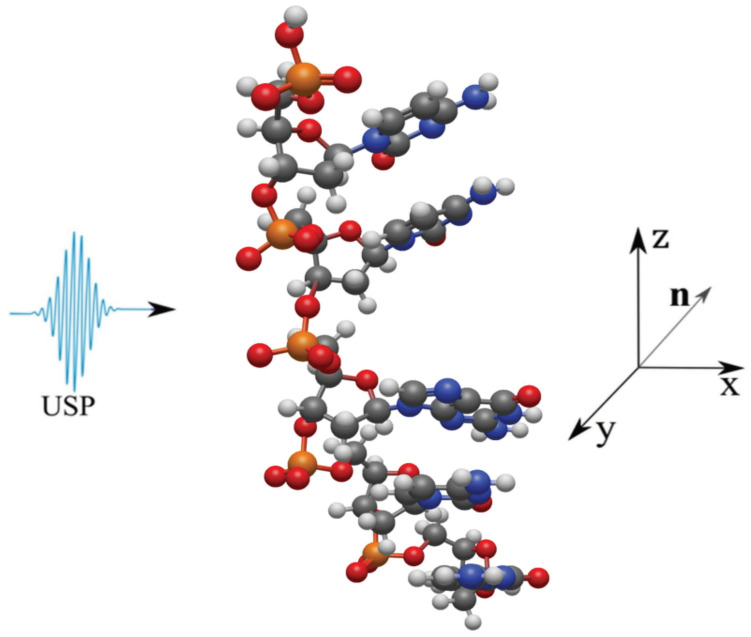
The USP is shown falling on a section of a single-stranded bending DNA. The colors of the atoms are the same as in Figure 2.

Let us consider the scattering of attosecond laser pulses on single-stranded DNA macromolecules shown in Figure 1. In this work, single-stranded DNA macromolecules are studied, since it is necessary to compare the scattering spectra of an undamaged DNA chain (Figure 1a) with a damaged one (Figure 1b,c). Obviously, if calculations on single-stranded DNA macromolecules differ from the same calculations using the previous theory, then the same will happen for double-stranded structures. We will also carry out calculations on the same structures in the case of using the previous XRD theory, i.e., where the pulse duration is considered infinitely long. It should be added that it is sufficient to study the duration of USP on scattering spectra on the non-periodic part of a single-stranded DNA molecule, represented in Figure 1 by the highlighted area. Indeed, taking into account a larger number of repeating regions only enhances the diffraction pattern from one part of it, i.e., the scattering spectra do not change qualitatively. Of course, calculations based on the complete DNA structure are interesting, but they are needed only for specific applied calculations. Our goal is to show that the use of Equation (1) when scattering attosecond pulses carries more error and it is necessary to take into account the pulse duration, and for this it is enough to perform calculation for a selected region of DNA that has a non-periodic structure. It should be added that such calculations were first carried out in the work in [19], where scattering spectra on DNA trinucleotides were studied and it was shown that, indeed, scattering spectra taking into account the pulse duration can differ greatly from the scattering spectra in the previously known theory. And the conclusions in the work in [19] indicate that such studies, but on more complex structures, need to be carried out. However, according to the results of the work in [19], it cannot be stated that the scattering spectra of the entire DNA molecule, taking into account the pulse duration, will differ from the previously known scattering theory, since trinucleotides are not a periodic structure of the entire DNA molecule; those considered in this work are the periodic structure parts, see highlighted areas Figure 1. Thus, we will show that the scattering spectra taking into account the pulse duration will differ from the scattering spectra in the previously known theory. Let us consider in more detail the three structures presented above, on which the USP falls, see Figure 2, Figure 3 and Figure 4.

It should be noted that scattering from spatially oriented structures is considered here, which is not the case in the TR-XRD experiment using the pump–probe technique. In the pump–probe method, the structures under study are fed randomly, oriented relative to the incident USP. To achieve this, it is necessary to average the obtained spectra over all possible angles of incidence of the ultrashort pulse. Visualization of studied processes in samples means new technologies for sample delivery. Such technologies currently include aerosol sample injection methods designed for individual particles and biomolecules, and liquid jets to produce continuous streams of nanocrystals [13,25]. All these techniques imply a random orientation of the supplied molecules. To calculate the scattering spectra, a single incident USP will be used, which corresponds to the pump–probe method. Indeed, in this technique, the sequence of X-ray USPs that “probe” the structure under study are located at a large time interval $\tau_{p}$ from each other, which is $\tau_{p}\gg \tau_{a}\gg \tau$.

Since attosecond pulses are considered here, the sudden perturbation approximation can be used to find the scattering spectra of ultrashort pulses. In this approximation, the theory of scattering of ultrashort pulses was developed in the works in [18,19,26]. In this approximation, it is assumed that the duration of an ultrashort pulse τ is many times shorter than the characteristic atomic time $\tau_{a}∼1$, i.e., $\tau \ll \tau_{a}$. Let us recall that for sufficiently light atoms, $\tau_{a}∼1$. Also, this theory is also suitable for longer pulses [27], including femtosecond ones. If we consider the USP to be spatially inhomogeneous, i.e., electromagnetic field strength $E\left(r,t\right)=E_{0}h\left(t-n_{0}r/c\right)$, where $E_{0}$ is the field amplitude, $h(t-n_{0}r/c)$ is an arbitrary function that determines the shape of the USP and *c* is the speed of light (in a.e. c≈137 ), then the scattering spectra (scattering energy and unit solid angle) of a Gaussian pulse $\tilde{h}\left(\omega \right)=\frac{\sqrt{\pi }}{\alpha }e^{-{\left(\omega -\omega_{0}\right)}^{2}/4\alpha^{2}}$ (α=1/τ, $\tilde{h}\left(\omega \right)$, this is the Fourier transform of h(x), $\omega_{0}$ is the carrier frequency of the pulse) can be represented as [18,26].

(2) $$\begin{array}{c} \frac{d\varepsilon }{d\Omega_{k}}=\frac{{\left[E_{0}n\right]}^{2}}{4c^{3}\alpha \sqrt{2\pi }}\left[\sum_{i=1}^{s}N_{e,i}N_{A,i}\left(1-\right|F_{i}\left(p_{0}\right){|}^{2})+\sum_{i,j=1}^{s}\gamma_{i,j}\left(p_{0},p_{\tau }\right)N_{e,i}N_{e,j}F_{i}\left(p_{0}\right)F_{j}^{*}\left(p_{0}\right)\right],\\ \gamma_{i,j}\left(p_{0},p_{\tau }\right)=\sum_{Ai,A^{{}^{\prime }}j}e^{-ip_{0}\left(R_{Ai}-R_{A^{{}^{\prime }}j}\right))}e^{-\frac{1}{2}{\left(p_{\tau }\left(R_{Ai}-R_{A^{{}^{\prime }}j}\right)\right)}^{2}}. \end{array}$$

where $N_{A,i}$ is the number of atoms of *i* type; $N_{e,i}$ is the number of electrons in the atom *i* type; $R_{Ai}$ is a radius vector specifying the position of an atom *i* of type with number $A_{i}$; the summation is carried out over all atoms $\left(A_{i},A_{j}\right)$ and over all types of atoms (i,j); $p_{0}=\frac{\omega_{0}}{c}\left(n-n_{0}\right)$ represents recoil momentum when light with frequency $\omega_{0}$ is scattered by a bound electron and $p_{\tau }=\frac{1}{c\tau }\left(n-n_{0}\right)$; and $F_{i}\left(p_{0}\right)=\frac{1}{N_{e,i}}\int \rho_{e,i}\left(r\right)e^{-ip_{0}r}d^{3}r$ is the form factor of the *i* atom of the variety with electron density $\rho_{e,i}\left(r\right)$. The electron density of the atoms of $\rho_{e,i}$ variety *i* will be chosen in the independent atom model [28]. In this case, we obtain $\rho_{e,i}\left(r\right)=\frac{N_{e,i}}{4\pi r}\sum_{k=1}^{3}A_{k,i}\alpha_{k,i}^{2}e^{-\alpha_{k,i}r}$, where $A_{k,i},\alpha_{k,i}$ are constant coefficients defined in [28]. It should be added that Equation (2) was obtained in the case of multi-cycle pulses, i.e., $\omega_{0}\tau \gg 1$. This case is precisely realized on well-known ultrashort-pulse X-ray sources, for example, Free-Electron Lasers (XFELs).

Let us add that if τ→∞ increases in Equation (2), then the well-known equation is obtained (1). Thus, Equation (2) contains characteristics responsible for the duration of USP τ. Indeed, if τ→∞, then the parameter $p_{\tau }=\frac{1}{c\tau }\left(n-n_{0}\right)\to 0$, and Equation (2) becomes proportional to τ (Fermi’s golden rule), and therefore coincides with Equation (1). In this case, it is especially interesting to understand the physical meaning of the $p_{\tau }$ momentum, since it is precisely this that is responsible for the main difference between Equation (2) and Equation (1). Its physical meaning is quite easy to understand if we consider that the incident USP is non-monochromatic, i.e., this pulse has a frequency dispersion proportional to 1/τ. This means that during USP scattering, a recoil momentum lying in the interval $∼p_{0}\pm p_{\tau }$ is transferred to atomic electrons. From Equation (2), namely the parameter $\gamma_{i,j}\left(p_{0},p_{\tau }\right)$, it is clear that the momentum $∼p_{0}\pm p_{\tau }$ can be transferred not to all electrons in the system under consideration, but only to those where the distance between electrons is of the order of cτ. In other words, due to frequency dispersion, it is not a plane wave of infinite extent that falls, but a wave packet of limited size ∼cτ; accordingly, only those electrons that are located inside the region of space ∼cτ are scattered together. As a result of this analysis, we can say that if we consider a structure whose asymmetrical part dimensions are comparable to or greater than ∼cτ, then the pulse duration will have a significant contribution to the scattering spectra, i.e., there will be a big difference between Equations (1) and (2). If attosecond pulses with τ∼1 and even an order of magnitude greater are used, then such systems can comprise various macromolecules, including DNA, RNA, various proteins, etc. However, the exact quantitative contribution can only be determined through direct calculations.

**Figure 5:**
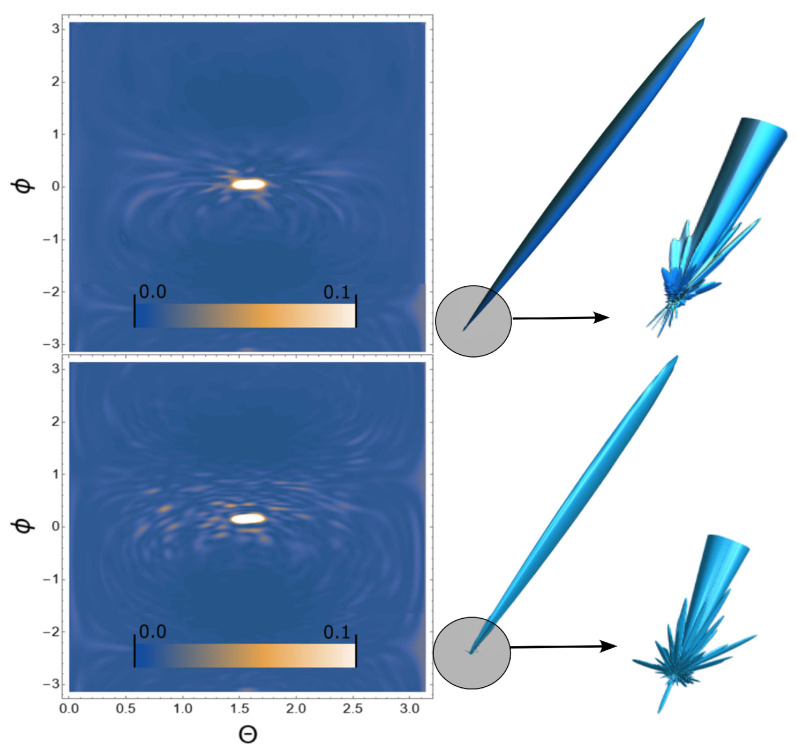
Scattering spectra of USP on a region of a single-stranded DNA molecule, see Figure 2: (upper part of the figures) calculated from Equation (2), (lower part of the figures) calculated from Equation (1). The spectra are presented as 2D (left) and 3D (right) graphs. Two-dimensional plots are presented in dimensionless units and normalized to the maximum value of the spectrum. Angles ϕ, θ are angles in the spherical coordinate system shown in Figure 2.

**Figure 6:**
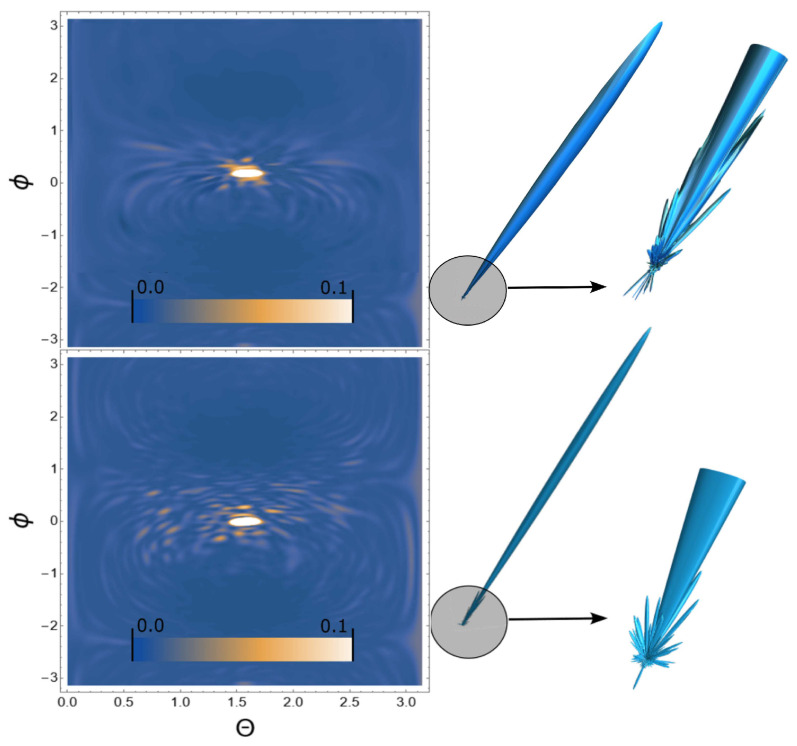
The same as in Figure 5, but the calculations were carried out for a section of a single-chain Nick molecule, see Figure 3.

**Figure 7:**
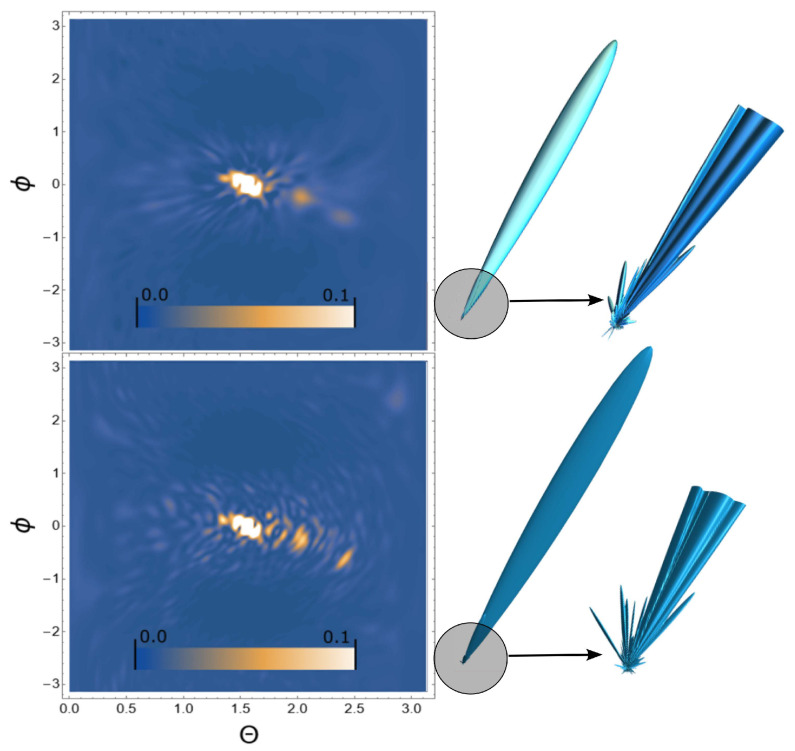
The same as in Figure 5, but the calculations were carried out for a section of a single-stranded bending DNA, see Figure 4.

Next, we consider the scattering of USPs on the systems presented in Figure 2, Figure 3 and Figure 4 and show that for such systems the use of Equation (1) introduces large errors. The results of calculations of scattering spectra using Equation (2) (top of figures) and using Equation (1) (lower part of the figures) are shown in Figure 5, Figure 6 and Figure 7. In the presented calculations, the incident USP on the systems under study is presented as shown in Figure 5, Figure 6 and Figure 7 with photon energy $\hbar \omega_{0}=7.46$ keV and pulse duration τ=1 (as). It should be added that the choice of USP duration is τ=1 as., which is purely conditional in order to better navigate the timeline. We can choose a longer duration in our calculations, about tens of τ∼10 (as); the results will be close, but more similar to the results of calculations using Equation (1). It should be added that USP durations of tens of attoseconds have already been implemented [29,30], and therefore our theory and calculations are implementable in practice.

It can be seen from Figure 5, Figure 6 and Figure 7 that the scattering spectra in the case of using Equations (1) and (2) are significantly different. The main part of the scattering in all cases occurs in the direction of the incident pulse, in figure these are light spots in the center. This scattering is of no interest to us, since there are no diffraction patterns in this scattering direction. Going beyond these limits, one can see that the diffraction pattern becomes very diverse and scattering occurs both forward and backward, i.e., throughout the entire region of space. These diffraction patterns are the “imprint” of the scattering substance. There is one basic pattern in the calculations when using Equation (1): there are more diffraction peaks, especially at large scattering angles. This is easy to explain based on the analysis in the previous section. When using Equation (1), the pulse duration is not taken into account in the calculations, which means the dimensions of such a pulse are infinitely large, i.e., this is a plane wave. Such a plane wave, incident on the system under study, interacts equally with all the electrons of the polyatomic system, which means there are more scattering centers. When using Equation (2), the pulse duration is taken into account, and therefore so is the size of the USP, which is ∼cτ. The size of our macromolecules are ∼100 (meaning linear size in atomic units of length), which is comparable to the size of USPs. This means that a USP incident on such a system does not interact equally with all electrons of such a structure, which leads to a decrease in scattering centers and, accordingly, diffraction peaks.

## 3. Materials and Methods

Scattering spectra were calculated using Wolfram Mathematica 11.2.0. The recommended minimum technical requirements for such calculations are the use of at least 4 gigabytes (GB) of RAM, a processor with a frequency of at least 2 gigahertz (GHz) with 4 cores, and at least 1 gigabyte of free hard disk space (HDD/SSD).

The method for calculating scattering spectra using Equation (2) is based on modeling these molecules using the known coordinates of atomic centers for the DNA molecule (Figure 1a) and the Nick molecule (Figure 1b), as well as bending at an arbitrary angle of the Nick molecule, see Figure 1c. The calculation was carried out by substitution into Equation (2) known coordinates of the centers of the atoms of these molecules.

## 4. Conclusions and Discussion

The main results obtained in this work are as follows:

1. The use of the previously known and widely used Equation (1) is unacceptable in the case of scattering of attosecond pulses on the structures under study, see Figure 2, Figure 3 and Figure 4.
2. To calculate the scattering spectra of attosecond pulses on the structures under study, it is necessary to use Equation (2), where the previous theory is a special case of the theory presented here.

These results are extremely important in the theory of time-resolved X-ray diffraction (TR-XRD). Indeed, to study the dynamics of processes occurring on the atomic and molecular time scale, i.e., where the characteristic time $\tau_{a}$ of such systems is comparable to tens of attoseconds, to study the dynamics of such systems it is necessary to use pulses with a duration $\tau \ll \tau_{a}$. When “decoding” the scattering spectra of such pulses, as shown above, the differences between the old and the presented theory can be very large, which will lead to an incorrect determination of the structure and dynamics of the system being studied. Thus, Equation (2) is more general in XRD (or TR-XRD) theory. The results obtained should be used to study the structures of DNA and RNA using attosecond pulses.

It should be noted that this work considered scattering from spatially oriented structures; despite this, similar calculations were carried out for randomly oriented molecules and all conclusions are the same as those presented above, i.e., averaging does not qualitatively change the scattering spectra.

## Data Availability

Upon request to corresponding author of this article.
